# Phenomenology of pregnancy and the ethics of abortion

**DOI:** 10.1007/s11019-017-9786-x

**Published:** 2017-07-01

**Authors:** Fredrik Svenaeus

**Affiliations:** Centre for Studies in Practical Knowledge, Södertörn University, 141 89 Huddinge, Sweden

**Keywords:** Ethics of abortion, Phenomenology, Lived body, Pregnancy, Obstetric ultrasound, Quickening, NIPT

## Abstract

In this article I investigate the ways in which phenomenology could guide our views on the rights and/or wrongs of abortion. To my knowledge very few phenomenologists have directed their attention toward this issue, although quite a few have strived to better understand and articulate the strongly related themes of pregnancy and birth, most often in the context of feminist philosophy. After introducing the ethical and political contemporary debate concerning abortion, I introduce phenomenology in the context of medicine and the way phenomenologists have understood the human body to be lived and experienced by its owner. I then turn to the issue of pregnancy and discuss how the embryo or foetus could appear for us, particularly from the perspective of the pregnant woman, and what such showing up may mean from an ethical perspective. The way medical technology has changed the experience of pregnancy—for the pregnant woman as well as for the father and/or other close ones—is discussed, particularly the implementation of early obstetric ultra-sound screening and blood tests (NIPT) for Down’s syndrome and other medical defects. I conclude the article by suggesting that phenomenology can help us to negotiate an upper time limit for legal abortion and, also, provide ways to determine what embryo–foetus defects to look for and in which cases these should be looked upon as good reasons for performing an abortion.

## Introduction

In this article I want to investigate the ways in which phenomenology could guide our views on the rights and/or wrongs of abortion. To my knowledge very few phenomenologists have directed their attention toward this issue (but see Mumford 2013), although quite a few have, indeed, strived to better understand and articulate the closely related themes of pregnancy and birth, most often in the context of feminist philosophy (Adams and Lundquist 2013; Bornemark and Smith 2015; Diprose 2002; Toledano 2016; Young 2005). After introducing the ethical–political contemporary debate about abortion I will move on to phenomenology in the context of medicine and how phenomenologists have viewed the human body not only as a biological organism but also as a “lived body”. I then turn to the issue of pregnancy and discuss how the embryo or foetus could *appear* for us, particularly from the perspective of the pregnant woman in quickening, and what such “showing up” may mean from an ethical perspective. The way medical technology has changed the experience of pregnancy—for the pregnant woman as well as for the father and/or other close ones—is discussed, particularly the implementation of early obstetric ultra-sound screening and blood tests (NIPT) for Down’s syndrome and other medical defects. I introduce the idea that screening measures for diseases/defects should only be strived for when the baby to be born with the disease/defect could be predicted to lead a life *considerably* more painful and alienated in terms of illness suffering than what is the case in a standard human life. I conclude the article by suggesting that phenomenology can help us to negotiate an upper time limit for legal abortion in absence of indications of medical defects. Phenomenology could also be helpful in providing ways to determine what embryo-foetus defects to look for and in which cases these should be looked upon as good reasons for performing an abortion, also in cases beyond the upper time limit for legal abortion in absence of indications of defects.

## The ethics of abortion

The ethics of abortion has been a battleground ever since the rise of bioethics in the late 1960s. The discussions on the wrongdoing of, or the right to, terminating pregnancy are heavily politicized and jam-packed with rhetoric, especially in the USA, but also in many other Western countries (Dworkin 1994: chapter two). The stalemate between pro-lifers and pro-choicers is more or less total and has often been presented as a war between religious and/or conservative and feminist and/or liberal ideologies and many different solutions have been established by the laws regulating abortion we find in the case of different countries (Warren 2009).

On the pro-life side you find the idea that the embryo-foetus is a *person* from very early on, perhaps even from day one. However, persons are most often understood as creatures possessing self-consciousness, language, memory and ability to plan their actions, so this is hardly a convincing view (DeGrazia 2005: Chap. 2). Even considering the fact that children in the normal case do not attain full personhood until about 4–5 years of age and that some children never do (because of defects) it remains unconvincing to assign personhood to a ball of cells only, even if these cells possess human DNA. A much more persuasive thought is that the embryo-foetus is protection worthy because it is a *potential* person (Gómez-Lobo 2004). While it is hard to deny that all embryos by way of their biology are human beings, the question whether they are also potential persons depends on they way one defines identity and potentiality in this context (Brown 2007). The genetic make up of the embryo, so the potentiality argument goes, directs its development from the very beginning, if it is given the opportunity to mature in its natural environment (meaning the uterus of a woman). Abortion is wrong, according to the pro-life argument, because it ends the life of a (potential) person.

On the pro-choice side you find the idea that the pregnant woman has the right to decide upon ending pregnancy, because the embryo-foetus is a part of her body. Persons have the right to decide upon what to do with their own bodies because the bodies *belong* to them. The idea of patient autonomy has been on the agenda of bioethics from the start and the right to legal abortion for every woman has been pursued as a part of this agenda—and other, political—movements. Women have the right to decide on issues that concern their reproductive life and the right to legal abortion is a part of this set of rights, as is the access to birth control or IVF. According to the pro-life argument, the choice if pregnancy should continue or not is for the pregnant woman to take, and nobody else’s business. If the foetus lives or dies is her decision, at least up until the point when it could survive outside her body by help of an incubator (Thomson 2006).

Could phenomenology offer a way to understand the ethical dilemmas surrounding abortion that would unlock the stalemate between pro-lifers and pro-choicers? Could it give us some ethical advice on in what situations abortion is a legitimate choice for a pregnant woman and in what situations it is not? I think it can, but first a disclaimer should be made. To do a phenomenological-ethical analysis of abortion is not the same thing as writing a law text proposal. Considerations beyond ethical arguments may play into political decision making in this and many other bioethics areas, and legitimately so (van der Burg 2009). Phenomenology is rather equipped to provide a *point of view* that better informs political decision- and law making than it can provide detailed regulations on its own. But this, I think, is characteristic for many valuable philosophical contributions to bioethics, not only for phenomenology. What phenomenology is able to offer is a perspective, which will make us see things slightly differently and, I hope, more comprehensibly in abortion ethics. Let us start with a brief introduction of what it means to do phenomenological analysis in questions regarding medical practice and health care.

## Phenomenology and medicine

The main topic of phenomenology of medicine so far has been *bodily* experiences of phenomena such as illness, pain, disability, giving birth, and dying (Meacham 2015; Toombs 2001; Zeiler and Käll 2014). Everybody has a body—a body which can be the source of great joy but also of great suffering to its bearer—as patients and health care professionals know more than well. The basic issue that the phenomenologist would insist on in this context is that not only does everybody *have* a body, everybody *is* a body. Not only can I experience my own body as an *object* of my experience—when I feel it or touch it or look at it in the mirror—but the body is also that which makes a person’s experiences possible in the first place. The body is my place in the world—the place where I am which moves with me—which is also the zero-place that makes space and the place of things that I encounter in the world possible (Gallagher 2005).

Normally what the phenomenologist refers to as “the lived body” remains in the background of our experience and our attention is instead focused on the things in the world that we are engaged with. In the works of Maurice Merleau-Ponty, to mention the most well know “body phenomenologist,” we find penetrating descriptions and conceptual analyses of such everyday experiences that are bodily in nature even though we are not focused upon the body: seeing, listening, walking, talking, dancing, reading, etc. (Merleau-Ponty 2012). In some situations, however, the body calls for our attention, forcing us to take notice of its existence in pleasant or unpleasant ways (Leder 2016; Slatman 2014). This experienced body can be the source of joy, as when we enjoy a good meal, do sports, have sex, or are just relaxing after a hard day of work. However, the body can also be the source of great sufferings to its bearer, when a person falls ill or is injured and experiences pain, nausea, fever, or difficulties to perceive or move (Aho and Aho 2008; Carel 2008).

When I am developing a headache, an example explored by Jean-Paul Sartre in *Being and Nothingness*, the phenomenologist would point out that the pain is not only a sensation experienced inside my head but something that invades my entire world experience (Sartre 1992; Svenaeus 2015). If the doctor examines my body with the help of medical technologies she may be able to detect processes going on in my brain and the rest of my body that are responsible for the headache, but they will never find my *experience*, the feel and meaning the pain has for me in my “being-in-the-world,” to speak in a phenomenological idiom invented by Martin Heidegger in his magnum opus *Being and Time* (1996). This difference between the first-person and the third-person perspective on the body is an important one. It makes it possible to explain not only how human experience is meaningful and material simultaneously, but also how the body belongs to a person in a stronger and more primordial sense than a pair of trousers, a car or a house do. A phenomenological take on embodiment is also helpful in understanding pregnancy and the way medical technologies are involved in maternal care and to these issues we now turn.

## Phenomenology and abortion

From the phenomenological point of view, questions concerning if, when, and on what indications abortion may be performed, must be answered by way of reference to the condition and situation of the pregnant woman, as well as the condition and situation of the embryo–foetus in its different developmental stages as they are revealed through the pregnant woman’s experiences and by way of medical investigations. As we will see, the embryo-foetus *shows up* in human experiences also before it reaches a stage of development in which we can assume that it has experiences of its own (Bornemark 2015). The main difference between the phenomenological and most pro-choice views on abortion in such an analysis will be that the body of the pregnant woman is not considered as her property, but as an embodied way of *being* that goes through drastic and significant changes in the process of pregnancy (Mumford 2013). The main difference between the phenomenological and most pro-life views on abortion will be that the being of the embryo–foetus must be considered from the perspective of the pregnant woman’s life as soon as implanted and not simply as a person-in-being taking residence in her body (Mackenzie 1992; Young 2005). As we will see, the focus on the embodied experience of pregnancy in developing arguments about abortion accords importance to the way the woman experiences the foetus presence inside her at a certain point of time (quickening) and the assumed experiences had by the foetus at a later point (sentience). Important is also the time point at which the foetus could survive in an incubator if pregnancy is ended (viability), because this underlines that at least from this time point we are dealing with two individuals and not one embodied experience only.

At least two different questions have to be dealt with in a phenomenological-ethical investigation of abortion. First, under what circumstances and possible time limits should it be permitted by law and made medically available for a woman to abort her embryo-foetus simply because she wishes to do so. Second, what other circumstances concerning the pregnancy (e.g. brought about by way of rape, fear for the woman’s life if continued) and state of the foetus (medical defects) would make it reasonable to extend an established time limit, and in these cases, how far should the benchmark be moved?

Let us begin with the question of legal abortion. The conditions and limits to qualify for such opportunities vary significantly in the laws of different countries. And the standards have often changed over time due to shifts in political majorities. The countries in Africa, South America, and South East Asia, generally do not allow abortion with the exception of rape or on medical grounds. On the other hand, abortion law is most often permissible in the countries of North America, Europe, North and West Asia, as well as Australia and New Zealand. However, among the countries that allow legal abortion, the circumstances concerning the procedure for a woman’s informed consent may differ. And as it stands, the upper time limit of legal abortion varies significantly from country to country, from 10 to 24 weeks of gestational time.

What circumstances have been taken into consideration in the political process of deciding how late a woman may decide upon abortion? Generally, countries that have a considerably vast time frame—USA, Great Britain, Singapore, Sweden, The Netherlands—refer to the *rights* of the individual woman to do as she pleases with her own body. Whereas countries that adopt stricter limitations—France, Finland, Denmark, Belgium, Portugal, Vietnam, to offer some examples—do so not on grounds of embryo rights, but, nevertheless, according to the *perspective* of the growing embryo–foetus (see my introduction to abortion ethics above). This perspective becomes acutely important in the stages when the foetus is suspected to *feel* things, such as pleasure or pain, or if it could possibly *survive* in an incubator. The question of when the foetus is equipped to feel pain is disputed and infused by the political debates surrounding abortion. As a consequence there is no scientific consensus on the issue, but week 22 appears to be a good estimation (Bellieni 2012). Babies born as early as week 22, or even late in week 21, have been saved in neonatal care (Edemariam 2007). It should be stressed, though, that babies born earlier than week 23 rarely survive, and that very-early prematurely born babies—as a rule—suffer from a variety of severe health problems.

The scientific and technological means to explore the life of the foetus and inventions that make it possible for prematurely born babies to survive outside the womb, affect our views on the acceptable upper time limit of abortion. If the right to abortion is defended on the grounds that the foetus is a part of a pregnant woman’s body, and *nothing else*, that the foetus may feel pain and possibly survive even should the pregnancy be terminated, appears to undermine the view that it is no more than a kind of extra organ belonging to the pregnant woman. However, should abortion be performed to save the life of the pregnant woman, or because the chances of the baby’s survival without severe defects are slim, the right (or even obligation) to perform abortion in week 22 or beyond could be defended on these grounds instead of the “my-body right” view. We will return to the issue of medically motivated abortions below.

## Quickening and bodily alienation

What other developmental milestones in the life of an embryo-foetus than sentience and viability should be taken into account when determining the time limits of legal abortion? From a phenomenological perspective, the most obvious one is the pregnant woman’s experiences of “quickening” (Bornemark 2015; Young 2005). The first sensations had by the pregnant woman of the foetus moving and kicking in her belly are, as a rule, felt in gestational week 18–20 (Sinha et al. 2012: 4). In the literature and on various webpages one finds reports of even earlier occurrences of quickening, so let us add two extra weeks (week 16) to be on the safe side. (In gestational weeks before week 16 it is probably very hard, if not impossible to distinguish foetal movements from bowel movements (gas).)

Quickening is a very significant occurrence because the woman can actually feel the presence of *another* human being inside her. This occurrence is very different from the experience of bodily alienation in illness that has been analysed by many phenomenologists (see the section on phenomenology and medicine above). Drew Leder has called such alienation the “dys-appearance” of the body in pain and illness in contrast to the *dis*appearance of the body enjoyed when the body stays in the background of our attentive field, which is the normal condition (Leder 1990: 69). The lived body, indeed, has a kind of background feel to it all the time, a way of being present that we can focus our attention upon by way of will. Yet, this way of sensing the different parts of the body, like when we do what is called a “body scan” in mindfulness training, is very different from the alienating force of the dysappearing body in pain. The healthy body offers a kind of primary being-at-home for us, which is turned into a not-being-at-home in illness (Gadamer 1996; Svenaeus 2009).

Iris Marion Young, who published her classic piece on the phenomenology of pregnancy already in 1983, argues that the experiences of pregnancy, including quickening, are not alienating in themselves (Young 2005). What alienates the life of pregnant and birthing women, according to Young, is the medical-technological gaze associated with the equipment of maternal care. Young’s perspective is typical of early phenomenology-of-medicine studies, assuming the medical perspective to be *inevitably* alienating and oppressive in nature, in contrast to a personally experienced, bodily transformation that would preserve the dignity and autonomy of the patient. In contrast to this view, I would argue that medical science and the attention of doctors and nurses are not necessarily alienating or oppressive for the patient (Slatman 2014; Svenaeus 2013). It is certainly neither of these when medical technologies provide means to limit severe suffering and save lives, such as is regularly the case in maternal care and birthing care. Notwithstanding this critique, I think Young and other feminist scholars are right in pointing towards the risks of *unnecessary* medicalizing pregnancy, and also in claiming that pregnancy, despite involving the experience of “an alien,” is not necessarily an alienating experience in this regard.

There is a clear difference between, for instance, the typical occurrence of morning sickness in early pregnancy and the events of quickening. The difference is between the experiences of the lived body as alien—in this case in nausea—and the experiences of *another living being* in my body. The foetus may to some extent be perceived as an unwelcome stranger—particularly if the pregnancy is unwanted—but in most cases, quickening is instead referred to as the first *contact* with the baby to come. To feel the foetus is to feel the *togetherness* of mother and child and this feeling is generally not referred to as alienating by the pregnant woman, but rather as the feeling of a different, and in some ways, fuller state of being (Bornemark 2015). Many feminists developing arguments about the right to legal abortion appear to miss, or, even, gravely misconstrue, the experiences of the pregnant woman by portraying it in terms of being chained to an alien when it is rather a matter of perceiving the successive arrival of a child. This is not only the case in Judith Jarvis Thomson’s famous thought experiment of waking up in the hospital back to back with an unconscious violinist, who has been plugged into your circulatory system (Thomson 2006), but also in cases of comparing the foetus to, for instance, a fish that has taken residence in the pregnant woman’s body (see the criticisms found in Mackenzie (1992) and Mumford (2013)).

Admittedly, this way of attempting to specify the phenomenological conditions of normal pregnancy runs the risk of underestimating the individual differences between pregnancies. If pregnancy is unwanted for the woman, and, especially, if it has been brought about by rape, the pregnant woman may feel the presence of the foetus to be exactly alien in nature. This may also be the case if the woman is afraid of how the new state of being will change her life, even if she does not wish to have an abortion, say if she is afraid of the pains of giving birth or of becoming a mother. Even so, quickening may in such cases also serve as a “counter alienating” experience in which the woman feels the foetus and exactly through this contact with a child to be accepts her pregnancy as a not entirely bad thing. In any case, a 16 weeks gestational time upper limit for legal abortion should provide plenty of time for early legal abortions in cases in which women do not want to continue with their pregnancies and give birth.

## The appearance of the foetus through obstetric ultrasound

The experience of quickening appears to be a strong candidate for setting an upper limit for legal abortion from the phenomenological point of view. This idea is not new; it appears to have proliferated in many pre-modern societies and cultural contexts that did not explicitly forbid early abortions (Dworkin 1994: 35 ff.). However, contemporary medical technologies have changed the way we establish the first contact with the child to come in comparison with pre-modern times. As a routine part of obstetric care, ultrasound pictures of the foetus are currently made for reasons of determining a more exact date of gestation, and to look for early signs of foetal abnormalities, such as Down’s syndrome. Obstetric ultrasounds are routinely performed in most developed countries of the world in the gestational interval of weeks 16–20 (and often earlier, see below). This time interval squares well with the first perceived movements of the foetus on part of the pregnant woman (quickening).

The differences between visual and inner-felt proof of foetal life are significant and it could be claimed that the pictures provided in the clinic are more of scientific documentation than contact with the baby to come. However, the routine of listening to the heartbeats of the foetus when viewing it on the screen and the provision of detailed, realistic pictures and videos by specialized commercial medical services, complicate the view that the ultrasound is only a medical-diagnostic tool. As a matter of fact, it could be argued that the pictures and videos of the foetus to be shared with others and put in the family album, are perceived as *more* real than the movements of the foetus experienced in quickening, even from the perspective of the pregnant woman. Vision, in comparison to the other senses (hearing, touch, taste and smell) has been privileged through our cultural history as offering the ultimate access to things in the world (“I see”), and this appears to apply even in the case of obstetric ultrasound and pregnancy. Ultrasound “opens up” the body of the pregnant woman, providing a new way of experiencing the presence of the foetus, for her, and for others (Mills 2011: 101–21).

As mentioned above, diagnostic ultrasound is carried out in gestational week 16–20 as a part of standard maternal care. This time interval squares well with the events of quickening and the technologically mediated contact with the foetus provided by the pictures, though it changes the experience of the pregnancy, does not change the view on the upper time limit of legal abortion from the phenomenological point of view. What complicates the matter of providing an upper time limit is the recent introduction in many countries of more or less routine ultrasound scans in a much earlier stage of pregnancy, roughly week 10–12. The reason for introducing these scans has been that ultrasound is less invasive than tests of foetal DNA for genetic disorders. For a long time, such tests have been recommended in high-risk pregnancies (e.g. high age of the mother to be), to test for Down’s syndrome (DS) and other medical defects. Because the sampling of amniotic fluid containing foetal DNA requires the insertion of a needle into the uterus, it carries a certain risk for miscarriage. Early ultrasounds were introduced to scan for embryo-foetal defects that could then be either confirmed or denied by way of amniocentesis (and/or maternal blood serum tests). The early ultrasounds, however, are presently in some countries not only offered in cases of high-risk pregnancies, but rather as a standard part of maternal care (in Denmark, for instance). In the countries where early ultrasound has been introduced, in combination with blood tests from the pregnant woman, the practice has lead to the performance of a large number of abortions when tests indicate a high risk for DS, which has subsequently radically reduced the number of babies born with this defect (Gordon 2015).

That a large number of babies with chromosomal or other congenital defects are never born because a life with such a defect is considered not to be worthwhile, and that the subsequent disabilities in question become rare or inexistent, might be problematic in itself. Our concern here, however, is how the early ultrasound affects our views, and arguments on legal abortion from the phenomenological point of view. If early ultrasound is not made use of as a routine test in maternal care, but only as a way of scanning high-risk pregnancies, it does not significantly change the analysis I have developed above. However, if early ultrasound is becoming part of standard maternal care, and is also being presented by the medical staff as a first opportunity to get to know the baby to come, the phenomenology of a pregnancy’s early-weeks will radically change That the same pictures made in week 10–12 to scan for medical defects routinely find their ways into the family albums of the prospective parents is deeply problematic, if we want to defend a right for women to legal abortion with an upper time limit of week 16 (the first possible experiences of quickening). Admitting that some parents to be may see the early ultrasound as a strictly medical procedure (no pictures saved, maybe not even looking at the screen) the standard way appears to be exactly to embrace it as way of making contact.

The child to come *appears* for the parents by way of the early ultrasound at a stage in pregnancy at which it was previously not identifiable as something distinct from the woman’s body. From the perspective of the doctors, nurses and midwifes, this is considered a good thing, since they think it makes it easier for the woman to embrace the pregnancy and take good care of the foetus. The problem from the vantage point of legal abortion, however, is that some foetuses in an early stage of development take on the ethical standing of children to be whereas others (that have not been scanned, or, are aborted as a consequence of the scan) are not considered to have any significant ethical standing. This is clearly ambivalent and the only way to remedy this inconsistency is, either, to adapt the upper time limit of legal abortion to the routine of the early ultrasound, or to only apply early ultrasound to high-risk pregnancies rather than use it as a standard way of establishing contact with the child to be. If we aim for the first, we should set the limit of legal abortion the same week that such a test is scheduled in maternity care. If we aim for the second, the last week of legal abortion should be the earliest week of quickening. The law would obviously have to state a specific week rather than refer to events in individual pregnancies.

## Abortion for medical reasons and the responsibility appeal

So far I have discussed how a phenomenological perspective on pregnancy will affect our views on legal abortion. Cases of pregnancies involving health risks for the pregnant woman open up for a permissive (or, even, injunctive) view on abortion in stages later than the weeks of gestation that we have discussed so far. If the life of the woman is threatened by the pregnancy, this overtrumps the perspective of the appearing, or even feeling foetus, as the foetus is not a person, but only a person in potential being. (The same argument could possibly also be used in cases of rape, in which the dignity, rather than the life, of the pregnant woman is threatened.) The remaining central reasons for abortion on grounds other than the woman’s wish not to have a child (at the present time) are of embryo-foetal defects identified through medical examinations and tests. Since it appears not only cruel, but also pointless to offer diagnostic tests without the option of abortion should the tests turn out positive, the challenging ethical question thus becomes: what tests should be made available or be made mandatory in maternal care. This brings us to the issue of if, and how, to choose the characteristics of children to be.

In his book, *The Imperative of Responsibility: In Search of an Ethics for the Technological Age*, the phenomenologist Hans Jonas offers the example of the new born, “whose mere breathing uncontradictably addresses an ought to the world around, namely, to take care of him” (Jonas 1984: 131). According to Jonas, the new born child, by way of its shear appearance, demands our attention and assistance in preserving his life and allowing him to prosper. The new born ushers in an ethical appeal to shoulder responsibility for his vulnerable and dependent being that is similar to the claim that originates from the face of the other in Levinasian ethics (Levinas 1991). This claim targets a temporal dimension by addressing the need to resume responsibility for the future and the generations to come (Jonas 1984: 136). Jonas’s main message in the book is the need to resume control of technologies that threaten the future of human life with their potential to destroy the ecological niches necessary for life on the planet (e.g. weapons of mass destruction, the plundering of natural resources and industrial pollution). However, his example of the new born child, who presents an “you ought to take responsibility for me,” is also interesting in the pre-birth context, even though Jonas himself never presented any consistent view on the rights or wrongs of abortion in the way he took a stand against cloning and genetic enhancement (Jonas 1987: 162–218).

As I have surveyed above, it could be argued that the foetus presents a similar, although perhaps weaker, claim of a need to be taken care of by presenting itself to the pregnant woman via quickening, or to her and other close responsibility-assumers in the ultrasound image. Such a claim would challenge the woman’s right to abortion, as well as other behaviours that would pose a risk to the health and life of the foetus. But what if the claim to be taken care of collides with knowledge about the future situation of the mother to be, or child to be, which makes the continuation of pregnancy to full-term appear *irresponsible*?

The responsibility to secure a future for the child when the pregnant woman judges her chances of taking good care of the new born to be slim or non-existent, for financial or other reasons, could possibly be handled by way of adoption, which means the challenge against the right to abortion in such cases of late abortions would still hold from the phenomenological angle. But what if the prediction of the future has to do with the baby’s health and possibility for flourishing rather than the mother’s? To knowingly give birth to a child with bodily defects that will lead to severe suffering and/or a radically shortened life, such as is the case in disorders such as anencephaly, Edward’s syndrome (Trisomy 18), muscular dystrophy, cystic fibrosis or Tay-Sachs disease, appears irresponsible and immoral, at least if the pregnancy could have been terminated when the foetus remained in a non-viable, or, even better, pre-quickening stage. The diagnostic tests for such genetic diseases are available precisely to spare future human beings of unnecessary suffering (Milunsky and Milunsky 2016).

This does not mean that such a human life with severe disease would in every case not be a life worth living—this depends on the severity of the disorder and the circumstances of the individual case— but it could be predicted to be *considerably* more painful and alienated in terms of illness suffering for the person in question. From a phenomenological perspective suffering could be viewed as a painfully attuned being-in-the-world separating a person from her goals and potentials in life (Svenaeus 2014). Such a mood (or combination of moods) involves painful experiences at different levels that are connected but are nevertheless distinguishable by being primarily about, firstly, the person’s embodiment, secondly, her engagements in the world together with others, and, thirdly, her core life-narrative values. The being-at-home or not-being-at-home of a person in a mood is interpreted as a being-in-the-world, which is also a being-as-a-body and a being-in-time (Heidegger 1996). Suffering, especially the sufferings brought on us by illness, is a bodily experience, but the alienating powers of suffering cover a territory that includes many kinds of life-world and self-interpretation issues.

Suffering is according to such a view in essence a feeling (a mood), but as such it has implications for and involves the person’s entire life: how she acts in the world, communicates with others, understands and looks upon her priorities and life goals. It is essential for medicine and bioethics to discern these different layers of suffering and how they are connected through the suffering-mood (Svenaeus 2014). Suffering-moods are typically intense and painful in nature, but they may also display a rather subconscious quality in presenting things in the world and my life as a whole in an alienating way. In the situation of living with severe diseases, like the ones mentioned above, it could be argued from the phenomenological point of view that the mood of the afflicted person is painful and the life alienated to a degree which makes such a life, if not worse than never being born, at least considerably worse than normal.

An argument in favour of abortion in this context would also include medical defects that tend to make a human life considerably worse when compared with normal circumstances, even if offering a rather large number of healthy years before facing a painful and deadly illness-suffering (Huntington’s disease, for instance). In such cases, the suffering will be considerable as soon as the person gets to *know* about her inevitable future falling-ill, and this suffering will also include knowledge about the risks of passing on the disease to children. The use of “considerable” in the argument above is admittedly vague, but it should at least lead us to assume grounds in support of abortion, also when the pregnancy has developed beyond the week limits we have considered for abortion on non-medical grounds. When the defect can be predicted to lead to severe suffering and a radically shortened life of the new born, the responsibility claim possibly transforms into an obligation to abort on part of the woman for the reason of avoiding undue suffering of a future person (DeGrazia 2005: Chap. 7).

## Prenatal diagnostic technologies and Down’s syndrome

The diagnostic tests on offer in maternal care scrutinize embryo-foetal DNA or other biomarkers found in amniotic fluid (or in the blood) of the pregnant woman, as well as bodily defects visible by way of the outer appearance of the foetus in forms of imaging such as ultrasound (Milunsky and Milunsky 2016). The currently most debated test in prenatal diagnostics is the ultrasound scan to detect Down’s syndrome (DS) by way of the NT procedure (nuchal translucency ultrasound scanning), followed up by blood serum and/or amniocentesis, also called combined ultrasound and blood (CUB) test. The questioning from an ethical point of view of ultrasound scanning for DS has been directed particularly towards early routine scanning beyond the boundaries of risk groups. Recently, the possibilities to detect genetic disorders by way of analysis of the very small amounts of foetal DNA that can be found in the blood of the woman from very early on in pregnancy (NIPT) has also been brought to the centre of attention (Dondorp et al. 2016). Such tests can be made to scan for genetic defects—or for genes associated with other characteristics than diseases—very early in pregnancy with very high accuracy. However, if the genetic disorders scanned for are rare, NIPT could nevertheless lead to a considerable number of false positives, if the test is used to scan the whole population of pregnant women as opposed to only risk groups. (NIPT is short for “non invasive prenatal testing,” which is potentially confusing, since a needle is injected to obtain the blood sample from the pregnant woman, but the use of “non invasive” is clearly meant in concern of the uterus in this case.)

The argument from critics against a recommended, more or less obligatory, early diagnostic test for DS in maternal care is that the tests lead to unnecessary abortions of foetuses at risk of carrying DS (especially when performed in an early stage of pregnancy) and that this is unfortunate because, first, these children may be unaffected (false positives), second, even if children have the syndrome, they may lead a good life, and, third, the tests and the ensuing abortions (carried out in about 90% of the positive cases) send the message to persons with DS and their families that these children are an unnecessary burden to society (Gordon 2015). In addition to these three concerns, one should mention that standard prenatal scanning for DS and other genetic disorders or diseases could lead to a less tolerant view in general on persons who are different from the healthy norm in our society in the future (Parens 2015).

Acknowledging these points of criticism, one should nonetheless take into consideration that even though a life with DS is not, as a rule, a life filled with suffering, it is without doubt a life that is more afflicted by medical problems than normal—a considerably higher risk of developing heart failure, neurological spasms, sleep apnoea, problems with speech, endocrine disturbances, gastrointestinal diseases and mental disorders are the most prevalent ones (Hickey et al. 2012). One should also remember that a life with DS, even if it does not involve major medical problems, is fraught with intellectual disabilities and that it is, in most cases, a life that is shorter than other human lives.

Admitting the medical problems to be real and in some case devastating to the life of persons with DS, the phenomenological perspective on suffering and its counterpart, flourishing, by way of concepts such as attuned being-in-the-world (see above), leave room for alternative ways of leading a non-alienated live with DS. Core life goals could be different than normal and so could the life narratives that DS persons identify with and embody together with family and friends. If possibilities for work and other meaningful activities are provided, such persons could flourish and contribute to our culture and society in many ways.

The main problem associated with judging if a life with DS is considerably more prone to suffering and/or considerably less prone to flourishing than a “standard” human life is that this varies from case to case. Some persons with DS appear to live happy and successful lives, whereas others suffer or are robbed of most normal life opportunities as a consequence of the syndrome. The prenatal tests cannot determine whether the child will be severely disabled or a case of “high performing” DS. However, the tests are able to determine with high accuracy—especially if we are considering ultrasound in combination with amniocentesis or NIPT—that the child will suffer from DS with standard complications in some degree of severity from the first day of birth. This is a main difference in comparison with other (prenatal) genetic tests that determine statistical *risks* for developing diseases at some point in life, tests that are already around and that will likely become increasingly common in the future with NIPT.

Most diseases are not single-gene disorders but multifactorial, involving many genes that determine risks for developing a disease in combination with environmental factors. We do not currently apply such diagnostic tests to determine risks that a child will develop, for instance, heart disease, various forms of cancer, ADHD or depression, at some point in life, because the risks associated with single genes or even combinations of many genes are too low and uncertain to motivate abortion, even if the tests would turn out positive. Perhaps the ultrasonic scanning for DS should rather be considered as such a low-risk test on the grounds that what the test should really determine if being relevant is the risk of developing DS with *major* medical problems and *severe* intellectual disabilities, not DS as such. If the current diagnostic test is to be considered as relevant or not depends on the size of the group of severe-suffering DS persons in relation to the group of mild-suffering DS persons. If the ratio is something like 20–80 we should probably not use the NT test as a standard procedure in maternal care. If the ratio is more like 80–20, the risk for severe-suffering DS is probably too high to not offer the test as a part of routine maternal care. I am currently unable to make the estimation, but I think this way of approaching the ethics of prenatal diagnostics for DS would be the best way to proceed. In making such an estimation, it should be kept in mind that the content moods or even happy moods, which persons with DS appear to standardly enjoy, clearly counts against considering such a life as fraught with suffering from the phenomenological perspective, despite the fact that important ways to flourish may be closed as a result of intellectual disabilities (Svenaeus 2014).

## The medical-technological gaze

Would a 50–50 ratio be enough to motivate scanning for DS in early pregnancy? Perhaps, but much depends on how criteria are set for the two groups of mild and severe DS. We will likely see much more risk-ratio and severity-issue discussions for a variety of diseases in the future if NIPT in early pregnancy is implemented as a standard part of maternal care. Since such risks, linked to certain genes, may also, in some cases, be “risks” of developing characteristics that we treasure—emotional sensitivity or intelligence in the case of depression and bipolar disorder, for instance—it is a development that should be closely monitored and ethically scrutinized. A broad implementation of NIPT would likely lead to a much larger number of abortions performed to avoid giving birth to babies with risks for developing diseases and/or carrying genetic defects of varying kinds. Such a development is ethically problematic for the different reasons that I have touched upon above: false-positive cases of abortions, abortions of embryos–foetuses that would have given rise to persons with lives that are not considerably worse than normal, stigmatizing effects for the persons who live with the diseases and disorders, and a less tolerant view in general on abnormalities in our society.

However, replacing early ultrasound scanning for DS with NIPT only, would have one significant advantage in light of the phenomenological argument about abortion developed above. The embryo–foetus tested by way of NIPT would not appear to the parents in the way the moving image of the foetus on the screen does and it would not result in any family album photographs. The blood test would not bring about any contact with a child to be, in the manner that ultrasound imaging currently does, but would rather be a medical-technological examination of the pregnant body, only. Nevertheless, one should not underestimate the way medical technologies, also in the cases not giving rise to spectacular images, is able to transform our perception and ways of thinking about what matters in a human life and what is normal. We are increasingly becoming objects of a medical-technological gaze, which we are making our own. Heidegger in the 1950s called this “Gestell”—an enframing of our world by science through which everything consequently shows up as calculable and usable (Heidegger 1977). Heidegger, in his essays on technology, mainly talked about forests, rivers and nuclear technology subjecting us to the *Gestell*, but the true extension of his analysis is the recent developments of gene technology, in which humankind itself is becoming the manipulated, not only the manipulator (Svenaeus 2013). Early ultra sound could be viewed as such an enframing, too, transforming pregnancy into production of normal babies, although the cultural meaning and use is arguably much more manifold than that, as I have discussed above.

It is obvious that many medical technologies, if brought and kept within the bounds of sound judgement and application, are too valuable to our lives to be abstained from, although they do force people to take a stand on and possibly change their attitudes towards their own lives and bodies (Svenaeus 2013). However, in the case of pregnancy this impact of medical technology has not only consisted in making pregnancy and birth safer for the mother and child, it also includes an impact on the relationship formed to the embryo–foetus and the risks of instrumentalizing human-reproductive issues. If children are chosen—or, rather, in the case of DS, not chosen—because of their genes instead of being received as persons in potential being, this could have negative effects on the parent–child relationship as such (Habermas 2003; Hauskeller 2014; Parens 2015). This is an important subject for so-called “human enhancement” studies, but a phenomenological analysis of the parent–child relationship and its transformation by way of future medical technologies making it possible to design babies lies beyond the scope of this article.

## Conclusions

The phenomenological analysis in the field of abortion ethics proceeds from the embodied perspective of the pregnant woman and from the imagined perspective of the embryo–foetus–new-born-child, informed by medical science and technologies. The main difference between the phenomenological and most pro-choice views on abortion in such an analysis will be that the body of the pregnant woman is not considered as her property, but as an embodied way of being that goes through drastic and significant changes in the process of pregnancy. The main difference between the phenomenological and most pro-life views on abortion will be that the being of the embryo–foetus must be considered from the perspective of the pregnant woman’s life and not simply as a person-in-being taking residence in her body.

The experience of quickening appears to be a strong reason from the phenomenological point of view for setting an upper time limit of legal abortion around week 16 gestational time. In quickening the pregnant woman feels the presence of another human being inside her and this as a rule is not an alienating event, but rather a part and process of a different form of embodied being, in contact with a child to be, which appeals for protection and support. In cases of being able to avoid giving birth to children who will suffer considerably more painful and alienated lives than normal, this responsibility for taking care of the child to be will be transformed into a responsibility to consider and/or have an abortion, also in cases of pregnancies continued beyond the weeks of quickening, or, even, in some cases, beyond the weeks of sentience and viability.

Diagnostic technologies, such as ultrasound imaging and various forms of genetic tests, do not only make it possible to abstain from having children who will suffer considerably more painful and alienated lives than normal, they also change the experience of pregnancy as such. The foetus appears to the pregnant woman and others by way of ultrasound images, videos and sounds, in some cases long before it will make itself known through movements in quickening. This must be taken into consideration in setting upper time limits of legal abortion.

In the case of Down’s syndrome (DS), the early tests by way of the NT procedure, followed up by blood serum and/or amniocentesis, and, in the future, NIPT, already has and probably will lead to less and less children with DS being born. Such a development is ethically problematic for the following reasons: abortions of embryos-foetuses that would have given rise to persons with lives that are not considerably worse than normal according to a phenomenological analysis, stigmatizing effects for the persons who live with DS, and a less tolerant view in general on abnormalities in our society. The last development is a clear indication that medical technologies do not only equip us with more choices regarding what type of children should be born, they also change our perception of what is a normal human life and what a life worth living looks like in the first place.
